# Impact of the gonococcal FC428 *penA* allele 60.001 on ceftriaxone resistance and biological fitness

**DOI:** 10.1080/22221751.2020.1773325

**Published:** 2020-06-04

**Authors:** Ke Zhou, Shao-Chun Chen, Fan Yang, Stijn van der Veen, Yue-Ping Yin

**Affiliations:** a Peking Union Medical College, Institute of Dermatology, Chinese Academy of Medical Sciences, Nanjing, People’s Republic of China; b National Center for STD Control, China Center for Disease Control and Prevention, Nanjing, People’s Republic of China; c Department of Microbiology and Parasitology, School of Medicine, Zhejiang University, Hangzhou, People’s Republic of China; d Department of Dermatology, School of Medicine, Sir Run Run Shaw Hospital, Zhejiang University, Hangzhou, People’s Republic of China; e State Key Laboratory for Diagnosis and Treatment of Infectious Diseases, Collaborative Innovation Center for Diagnosis and Treatment of Infectious Diseases, School of Medicine, The First Affiliated Hospital, Zhejiang University, Hangzhou, People’s Republic of China

**Keywords:** *Neisseria gonorrhoeae*, FC428, *penA* 60.001, ceftriaxone, biological fitness

## Abstract

Global dissemination of the *Neisseria gonorrhoeae* ceftriaxone-resistant FC428 clone jeopardizes the currently recommended ceftriaxone-based first-line therapies. Ceftriaxone resistance in the FC428 clone has been associated with the presence of its mosaic *penA* allele 60.001. Here we investigated the contribution *penA* allele 60.001 to ceftriaxone resistance and its impact on biological fitness. Gonococcal isolates expressing *penA* allele 60.001 and mosaic *penA* allele 10.001, which is widespread in the Asia-Pacific region and associated with reduced susceptibility to ceftriaxone and cefixime, were genetic engineered to exchange their *penA* alleles. Subsequent antimicrobial susceptibility analyses showed that mutants containing *penA* 60.001 displayed 8- to 16-fold higher ceftriaxone and cefixime minimal inhibitory concentrations (MICs) compared with otherwise isogenic mutants containing *penA* 10.001. Further analysis of biological fitness showed that *in vitro* liquid growth of single strains and in the competition was identical between the isogenic *penA* allele exchange mutants. However, in the presence of high concentrations of palmitic acid or lithocholic acid, the *penA* 60.001-containing mutants grew better than the isogenic *penA* 10.001-containing mutants when grown as single strains. In contrast, the *penA* 10.001 mutants outcompeted the *penA* 60.001 mutants when grown in competition at slightly lower palmitic acid or lithocholic acid concentrations. Finally, the *penA* 60.001 mutants were outcompeted by their *penA* 10.001 counterparts for *in vivo* colonization and survival in a mouse vaginal tract infection model. In conclusion, *penA* allele 60.001 is essential for ceftriaxone resistance of the FC428 clone, while its impact on biological fitness is dependent on the specific growth conditions.

## Introduction

*Neisseria gonorrhoeae* causes the widespread bacterial sexually transmitted disease gonorrhoea, which is predicted to have an annual global incidence of 87 million new cases [1]. Infections commonly manifest as urethritis or cervicitis, but asymptomatic infections of the cervix or pharynx are very frequently observed [2,3]. These untreated infections occasionally result in severe complications, including ectopic pregnancies and pelvic inflammatory disease, and they are a major source for transmission of *N. gonorrhoeae* [4]. *N. gonorrhoeae* is a multidrug-resistant pathogen that has developed resistance against all previously used antimicrobial therapies [5]. Current first-line treatment guidelines generally recommend ceftriaxone as a single drug therapy or ceftriaxone in combination with azithromycin as a dual therapy. However, many countries have reported increasing incidences of azithromycin resistance, including high-level azithromycin [6–8], and therefore the inclusion of azithromycin in the dual therapy has recently become under scrutiny [9]. Furthermore, gonococcal susceptibility to ceftriaxone is decreasing in many countries [8,10–12] and ceftriaxone treatment failures are increasingly reported globally [13–18]. Importantly, while initially ceftriaxone treatment failures were attributed to sporadic infections of unrelated strains containing mosaic *penA* alleles providing ceftriaxone resistance [17,19–22], in recent years many of the reported ceftriaxone treatment failures are the result of the FC428 clone identified in 2015 in Japan [23]. This clone contains the mosaic *penA* allele 60.001 and has successfully transmitted on a global scale, with reported cases in Japan [24], China [25–27], Denmark [28], Canada [29], Australia [30], Ireland [31], UK [32], and France [14]. In addition, incidences where this *penA* 60.001 allele has transferred to unrelated strains and subsequently caused treatment failure have also been reported [33]. In recent years, the FC428 clone has widely transmitted throughout China, since cases have been reported from many geographically distinct regions [34], and its incidence also appears to be rapidly increasing [35].

The mosaic *penA* allele 60.001 contains the A311 V and T483S polymorphisms that were considered as essential mutations in the high-level ceftriaxone-resistant strains HO41, A8804 and GU140106 isolated previously in Japan and Australia [21,22,36], although additional I312M, F504L, N512Y and G545S polymorphisms associated with reduced cephalosporin susceptibility in mosaic *penA* alleles [37,38] are also present in *penA* allele 60.001. Importantly, the mosaic alleles *penA* 37 (HO41) and *penA* 42 (F89), which provide high-level ceftriaxone resistance, incur a biological fitness cost. Cloning of these *penA* alleles in unrelated gonococcal isolates had a negative impact on *in vitro* growth and these mutants were outcompeted by their otherwise isogenic wild-type strains for colonization in a mouse model of infection [39]. The negative impact of these *penA* alleles on biological fitness might explain why these ceftriaxone-resistant strains have thus far remained sporadic and have not widely transmitted. However, this might have changed with the occurrence of the ceftriaxone-resistant FC428 clone, which has transmitted globally. The *penA* allele of this strain might not incur a severe fitness cost as observed for other ceftriaxone-resistant *penA* alleles, which could explain its successful global transmission. Therefore, the aim of the present study was to investigate the impact of *penA* allele 60.001 on biological fitness during *in vitro* growth in cultures and *in vivo* in a mouse model of infection.

## Materials and methods

### Bacterial strains, mutants and culture conditions

*N. gonorrhoeae* strains ATCC49226, SZ20 (*penA* 60.001, *mtrR* 1, *ponA* 1) [26], SRRSH78 (*penA* 10.001, *mtrR* 1, *ponA* 1) [8] and their derivatives were cultured on GC agar (Oxoid Ltd., Basingstoke, UK) containing 1% (v/v) Vitox (Oxoid Ltd., Basingstoke, UK) at 37°C and 5% CO_2_ and stored in GC broth containing 15% glycerol (Biosharp, Hefei, China) at −80°C. The streptomycin-resistant derivatives of strains SZ20 and SRRSH78 were selected on GC agar containing 1% Vitox and streptomycin (BBI, Shanghai, China). These streptomycin-resistant derivatives were named SZ20-*penA*60 and SRRSH78-*penA*10, respectively, and contained their original *penA* alleles, but were given this name for clarity throughout the study about phenotypes associated with *penA* alleles. Strains SZ20-*penA*60.001 and SRRSH78-*penA*10.001 were genetically engineered using the dominant streptomycin-susceptible *rpsL* gene to exchange their *penA* alleles without leaving a selection marker [40]. Fragments of *penA* 60.001 and *penA* 10.001 were amplified from the SZ20 and SRRSH78 genomes, respectively, using primers pen*A*-F (GCGAGCTCGCAGTGGGAGGCTGAGAT), *penA*-R (GCTCTAGACGCTGGTTACGACGACTTTAT), *penA*-F2 (GCAGATCTCCGTCTTAATCCGAGTATCA) and *penA*-R2 (GCGTCGACGCAACCGAATACGCACCAT) and cloned into vector pUC57-*kanR*-*rpsL* [40], thereby generating vectors pUC57-*penA*60 and pUC57-*penA*10. These vectors were subsequently linearized and transformed into strains SZ20-*penA*60 and SRRSH78-*penA*10 to generate SZ20-*penA*10 and SRRSH78-*penA*60. The chloramphenicol-resistant derivatives SZ20-*penA*60-*catA2*, SZ20-*penA*10-*catA2*, SRRSH78-*penA*10-*catA2*, and SRRSH78-*penA*60-*catA2* were generated with the vector pUC57-*lctP-catA2-aspC* [41], which inserts the chloramphenicol-resistant gene *catA2* in the unrelated convergent *lctP*-*aspC* locus. The kanamycin-resistant derivatives SZ20-*penA*60-*kanR*, SZ20-*penA*10-*kanR*, SRRSH78-*penA*10-*kanR*, and SRRSH78-*penA*60-*kanR* were generated with the vector pUC57-*lctP-kanR-aspC* [42], which inserts the kanamycin-resistant gene *kanR* in the *ctP*-*aspC* locus.

### Ceftriaxone and cefixime susceptibility assays

*N. gonorrhoeae* strains were tested for ceftriaxone and cefixime susceptibility using the agar dilution method according to WHO guidelines and *N. gonorrhoeae* ATCC49226 was included for quality control. Overnight grown bacteria were suspended into GC broth containing 1% Vitox and droplets containing 10^4^ CFU were spotted onto GC agar plates containing 1% Vitox and a twofold dilution series of ceftriaxone or cefixime. Plates were incubated for 24 h at 37°C and 5% CO_2_ and the minimal inhibitory concentration (MIC) was determined as the lowest concentration at which no growth was observed.

### Liquid growth and in vitro competition assays

Overnight grown bacteria were suspended in 12 mL GC broth containing 1% Vitox at an optical density (OD_600_) of 0.025. Cultures were incubated at 37°C and 200 rpm and samples were taken every two hours for OD_600_ measurements. For growth in the presence of fatty acids or bile, 2 mg/L (SZ20 derivatives) or 4 mg/L (SRRSH78 derivatives) palmitic acid (Aladdin, Shanghai, China), or 10 mg/L (SZ20 derivatives) or 85 mg/L (SRRSH78 derivatives) lithocholic acid (Aladdin, Shanghai, China) were added. For competition assays, overnight grown isogenic strains containing *penA* 60.001 and *penA* 10.001 and expressing different selection markers were mixed at equal numbers and suspended in 12 mL GC broth containing 1% Vitox at an OD_600_ of 0.025. Culture was incubated at 37°C and 200 rpm and every two hours samples were taken, serially diluted and plated onto GC agar containing 1% Vitox and 100 mg/L kanamycin (Inalco SpA, Milano, Italy) or 7.5 mg/L chloramphenicol (Inalco SpA, Milano, Italy). Plates were incubated for 24–48 h at 37°C and 5% CO_2_ and colonies were enumerated. For competition assays in the presence of fatty acids or bile, 1.25 mg/L (SZ20 derivatives) or 1.5 mg/L (SRRSH78 derivatives) palmitic acid, or 5 mg/L (SZ20 derivatives) or 60 mg/L (SRRSH78 derivatives) lithocholic acid was added.

### Spot assays

Overnight grown bacteria were suspended in GC broth containing 1% Vitox and 5 μL droplets of a tenfold dilution series were applied on GC agar containing 1% Vitox and on GC agar containing 1% Vitox and 12 mg/L (SZ20 derivatives) or 160 mg/L (SRRSH78 derivatives) palmitic acid, or 10 mg/L (SZ20 derivatives) or 70 mg/L (SRRSH78 derivatives) lithocholic acid. Plates were incubated for 24–48 h at 37°C and 5% CO_2_ and colonies were enumerated. The growing fraction of bacteria on the fatty acid/bile-supplemented agar plates was expressed relative to the growing fraction on agar plates without fatty acid/bile.

### *In vivo* competition assays in a mouse vaginal tract model of infection

Competition assays in a mouse vaginal tract infection model were performed as described previously [40,41,43]. Dioestrus stage female BALB/c mice (Shanghai SLAC Laboratory Animal Company, Shanghai, China) at six to eight weeks of age were injected subcutaneously with 0.1 mg of β-estradiol (Aladdin, Shanghai, China) in sesame oil (Sigma-Aldrich Co., St Louis, USA) on days –2, 0 and 2. In addition, mice also received two doses of 0.6 mg vancomycin (Meilunbio, Dalian, China) and 1.2 mg streptomycin every day and drinking water was spiked with 0.4 g/L trimethoprim (Meilunbio, Dalian, China). Mixed bacterial suspensions containing equal numbers of strain SZ20-*penA*60-*catA2* and strain SZ20-*penA*10-*kanR* or equal numbers of strain SRRSH78-*penA*10-*kanR* and strain SRRSH78-*penA*60-*catA2* were formulated in PBS with 0.5 mM CaCl_2_ (Sigma-Aldrich Co., St Louis, USA), 1 mM MgCl_2_ (Sigma-Aldrich Co., St Louis, USA) and 1% (w/v) gelatin (Aladdin, Shanghai, China) and inoculated intravaginally on day 0 at a total dose of 2×10^7^ CFU. Daily bacterial load in the vaginal tract were monitored by swabbing and plating on GC agar containing with 1% Vitox, 3 mg/L vancomycin, 7.5 mg/L colistin (Meilunbio, Dalian, China), 2.8 mg/L nystatin (Meilunbio, Dalian, China), 5 mg/L trimethoprim, 100 mg/L streptomycin and 100 mg/L kanamycin or 7.5 mg/L chloramphenicol. The competition index (CI) was calculated as (*penA*10/*penA*60)_output_/(*penA*10/*penA*60)_input_. All animal experiments were approved by the Zhejiang University Animal Care and Use Committee under project license number ZJU2015-032-01. Procedures followed the guidelines of the Administration of Affairs Concerning Experimental Animals of the People’s Republic of China and adhered to the principles of the Declaration of Helsinki.

## Results

### Contribution of *penA* allele 60.001 to cephalosporin resistance

Ceftriaxone resistance in the FC428 clone has been widely attributed to the presence of *penA* allele 60.001, although its specific contribution has never been experimentally verified. Therefore, *penA* allele replacement mutants were generated for *N. gonorrhoeae* strains SZ20 and SRRSH78, which contain *penA* allele 60.001 and *penA* allele 10.001, respectively. Strain SZ20 was isolated in 2016 from a patient in Suzhou [26], and is closely related to the FC428 clone because besides an identical *penA* allele, it also shows identical MLST (ST1903), NG-MAST (ST3435) and NG-STAR (ST233) sequence types. Strain SRRSH78 was isolated in 2016 from a patient in Hangzhou [8], and contains *penA* allele 10.001. This *penA* allele is the most widespread mosaic *penA* allele in China and other Asia-Pacific countries and is generally mostly associated with low-level cefixime resistance or reduced susceptibility (MIC ≤0.25 mg/L) [8,44,45]. Furthermore, *penA* allele 10.001 contains the I312M, F504L, N512Y and G545S polymorphisms associated with reduced cephalosporin susceptibility, similar to *penA* allele 60.001, but not the A311 V and T483S polymorphism associated with high-level resistance. Ceftriaxone and cefixime susceptibility analysis showed that strain SZ20 was indeed resistant against ceftriaxone (MIC=0.5 mg/L) and cefixime (MIC=2 mg/L), while strain SRRSH78 was susceptible to ceftriaxone and low-level resistant to cefixime (Table 1). Importantly, isogenic strains in which the *penA* 60.001 and 10.001 alleles were exchanged showed an inversion of susceptibility, highlighting that *penA* allele 60.001 provides higher resistance to cephalosporins compared with *penA* allele 10.001 The strains containing *penA*60 showed eight- to sixteen-fold higher ceftriaxone MIC values compared with isogenic strains containing *penA*10. Similarly, *penA*60 strains showed eightfold higher cefixime MIC values compared with *penA*10 strains. These results highlight the important contribution of *penA* allele 60.001 to both ceftriaxone and cefixime resistance.

**Table 1.** Ceftriaxone and cefixime susceptibility of gonococcal wild-type strains and penA allele exchange mutants.

Strain	MIC (mg/L)	
	Ceftriaxone	Cefixime
ATCC49226	0.016	0.03
SZ20	0.5	2
SZ20-*penA*60	0.5	2
SZ20-*penA*10	0.03	0.25
SRRSH78	0.06	0.25
SRRSH78-*penA*10	0.06	0.25
SRRSH78-*penA*60	0.5	2

### Impact of *penA* allele 60.001 on in vitro biological fitness

**Figure 1.**
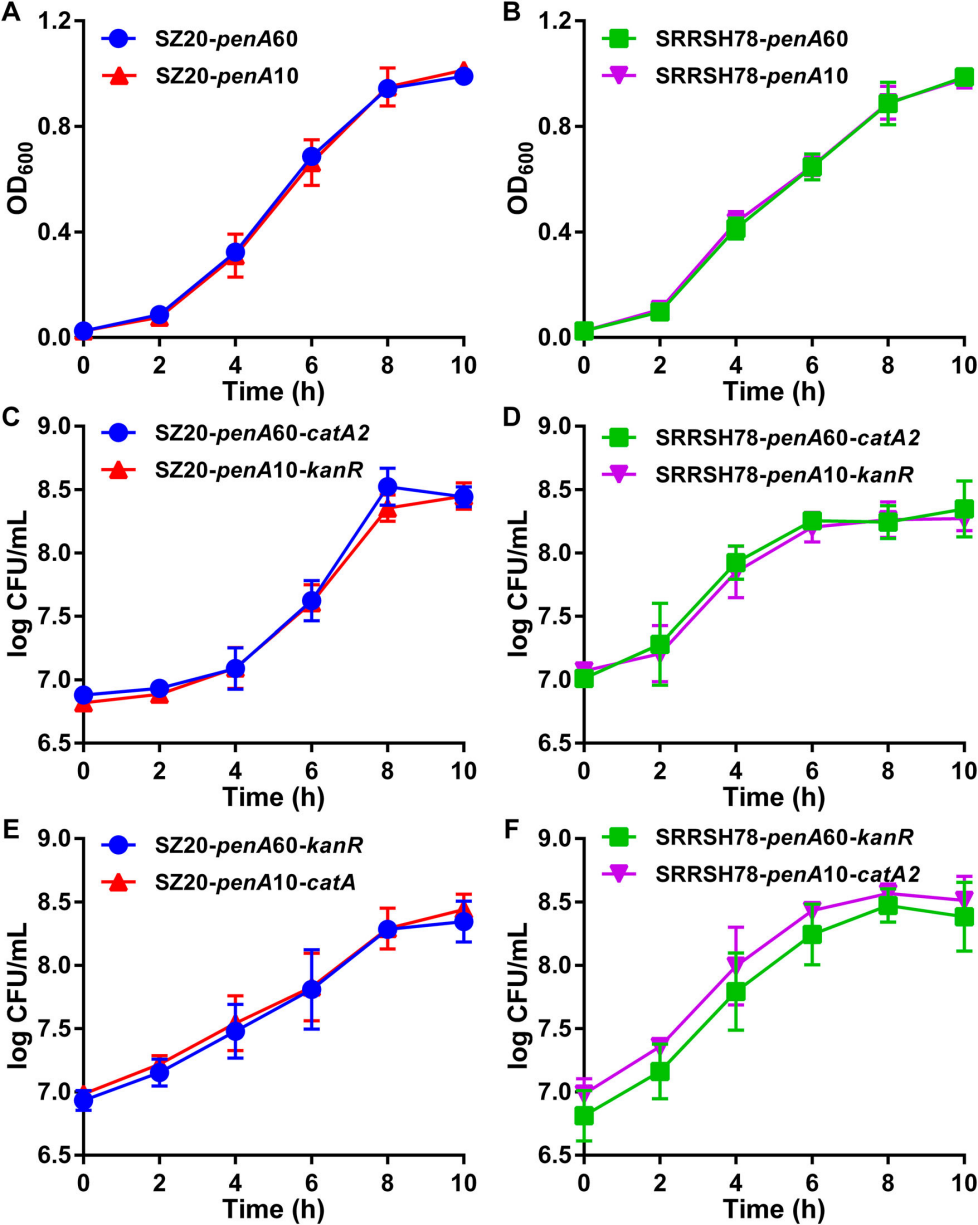
*In vitro* growth curves of the gonococcal *penA*60/*penA*10 allele exchange mutants in liquid culture. (A) Growth of single-strain SZ20 *penA* mutants (SZ20-*penA*60 and SZ20-*penA*10) determined by absorbance measurements (OD_600_) in liquid culture. (B) Growth of single-strain SRRSH78 *penA* mutants (SRRSH78-*penA*60 and SRRSH78-*penA*10) determined by absorbance measurements (OD_600_) in liquid culture. (C) Growth of strains SZ20-*penA*60-*catA2* and SZ20-*penA*10-*kanR* in competition in liquid culture determined by CFU counts on selective agar plates. (D) Growth of strains SRRSH78-*penA*60-*catA2* and SRRSH78-*penA*10-*kanR* in competition in liquid culture determined by CFU counts on selective agar plates. (E) Growth of strains SZ20-*penA*60-*kanR* and SZ20-*penA*10-*catA2* in competition in liquid culture determined by CFU counts on selective agar plates. (F) Growth of strains SRRSH78-*penA*60-*kanR* and SRRSH78-*penA*10-*catA2* in competition in liquid culture determined by CFU counts on selective agar plates. The graphs represent the average and standard deviation of three biological independent experiments.

**Figure 2.**
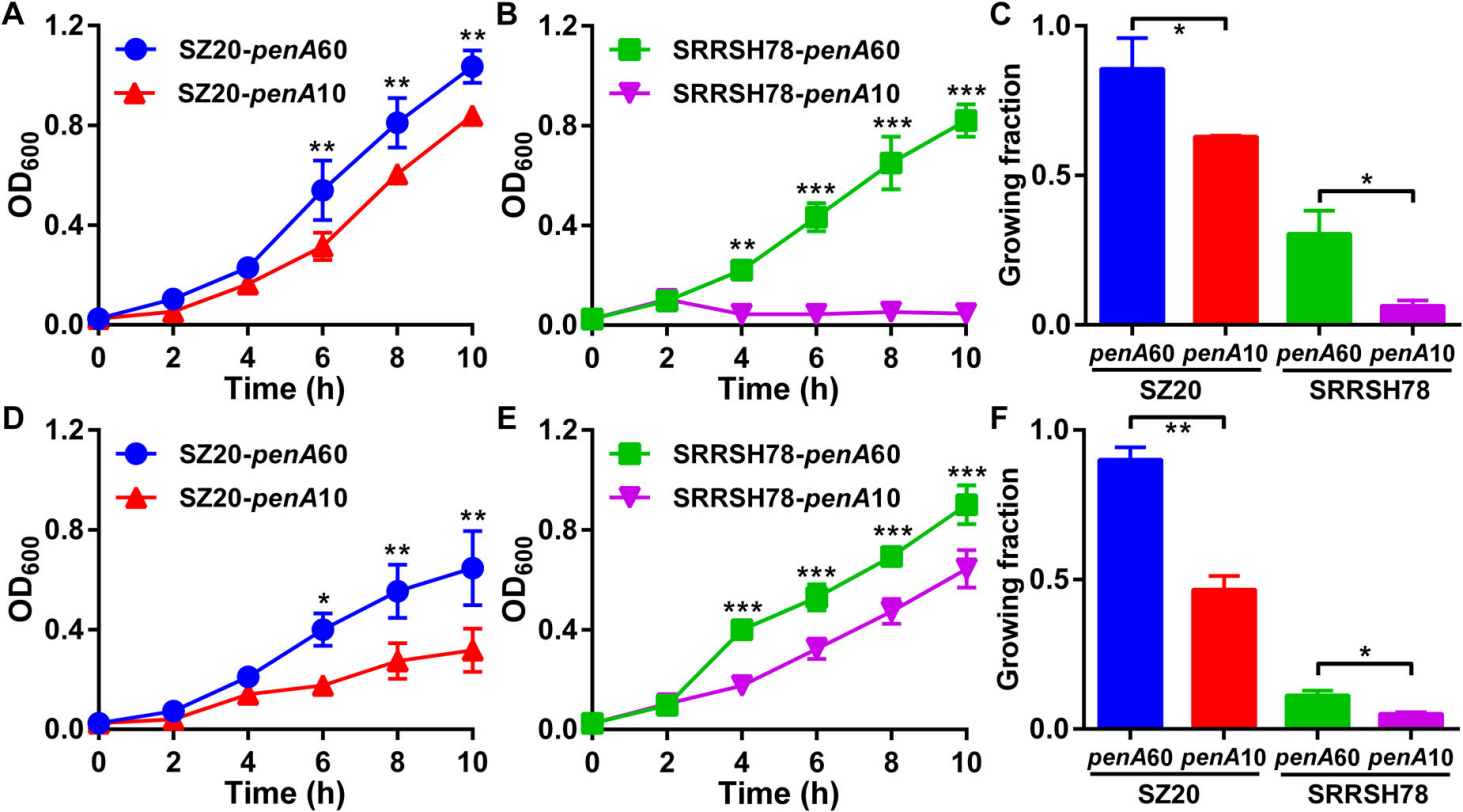
*In vitro* growth of the gonococcal *penA*60/*penA*10 allele exchange mutants in the presence of palmitic acid and lithocholic acid. (A) Growth curves determined by OD_600_ measurements of SZ20-*penA*60 and SZ20-*penA*10 in the presence of 2 mg/L palmitic acid. (B) Growth curves determined by OD_600_ measurements of SRRSH78-*penA*60 and SRRSH78-*penA*10 in the presence of 4 mg/L palmitic acid. (C) Growing fraction of the *penA* mutants on agar plates containing 12 mg/L (SZ20 derivatives) or 160 mg/L (SRRSH78 derivatives) palmitic acid relative to growth on control agar plates. (D) Growth curves determined by OD_600_ measurements of SZ20-*penA*60 and SZ20-*penA*10 in the presence of 10 mg/L lithocholic acid. (E) Growth curves determined by OD_600_ measurements of SRRSH78-*penA*60 and SRRSH78-*penA*10 in the presence of 85 mg/L lithocholic acid. (F) Growing fraction of the *penA* mutants on agar plates containing 10 mg/L (SZ20 derivatives) or 70 mg/L (SRRSH78 derivatives) palmitic acid relative to growth on control agar plates. The graphs represent the average and standard deviation of three biological independent experiments. Significant differences between the *penA*60/*penA*10 mutants at corresponding time-points were identified by Student’s two-tailed unpaired *t*-test (GraphPad Prism). *P<0.05; **P<0.01; ***P<0.001.

**Figure 3.**
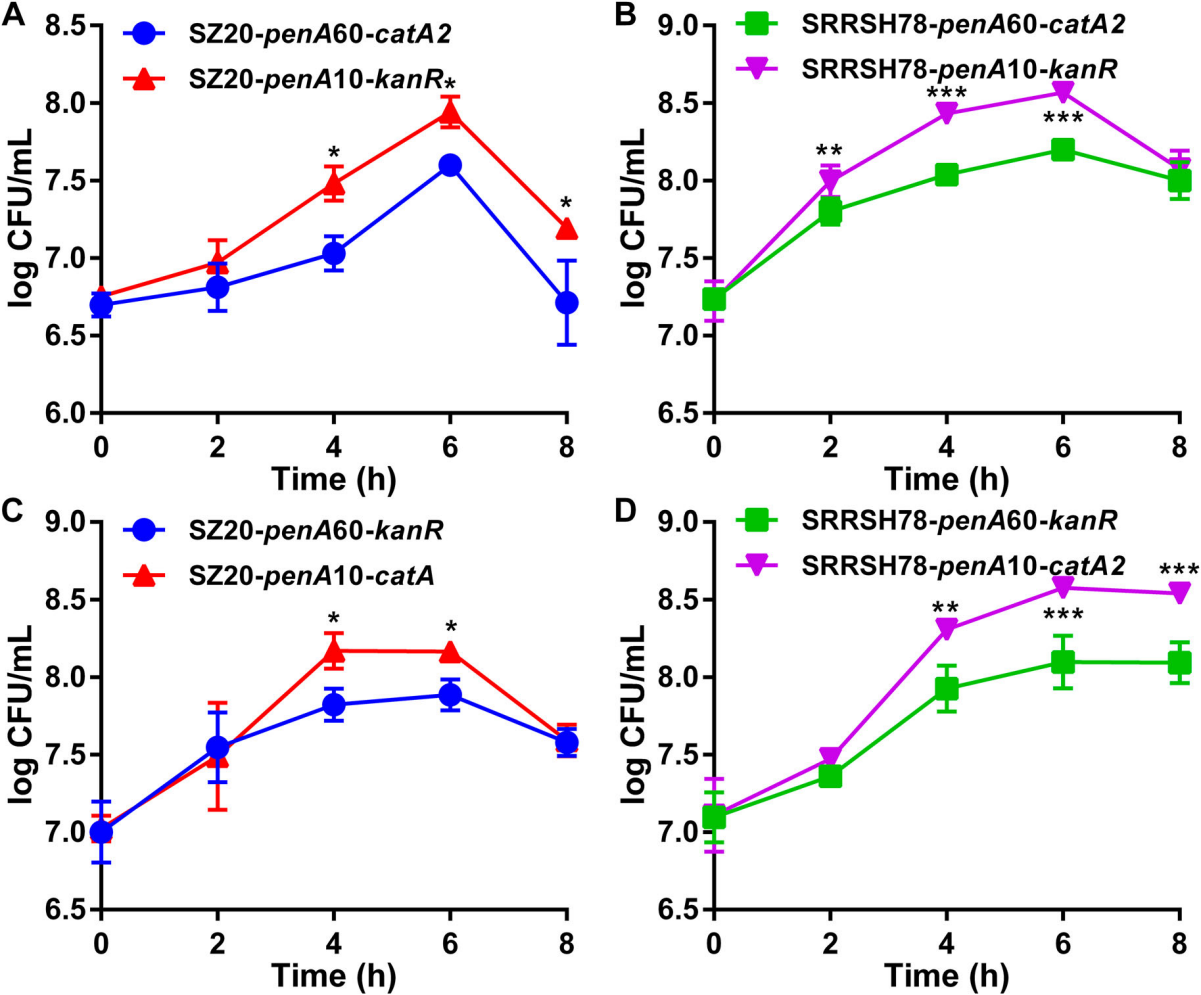
*In vitro* competition assays of the gonococcal *penA*60/*penA*10 allele exchange mutants in the presence of palmitic acid. (A) Growth of strains SZ20-*penA*60-*catA2* and SZ20-*penA*10-*kanR* in competition in liquid culture containing 1.25 mg/L palmitic acid. (B) Growth of strains SRRSH78-*penA*60-*catA2* and SRRSH78-*penA*10-*kanR* in competition in liquid culture containing 1.5 mg/L palmitic acid. (C) Growth of strains SZ20-*penA*60-*kanR* and SZ20-*penA*10-*catA2* in competition in liquid culture containing 1.25 mg/L palmitic acid. (D) Growth of strains SRRSH78-*penA*60-*kanR* and SRRSH78-*penA*10-*catA2* in competition in liquid culture containing 1.5 mg/L palmitic acid. Competitive growth was determined by CFU counts on selective agar plates. The graphs represent the average and standard deviation of three biological independent experiments. Significant differences between the *penA*60/*penA*10 mutants at corresponding time-points were identified by Student’s two-tailed unpaired *t*-test (GraphPad Prism). *P<0.05; **P<0.01; ***P<0.001.

**Figure 4.**
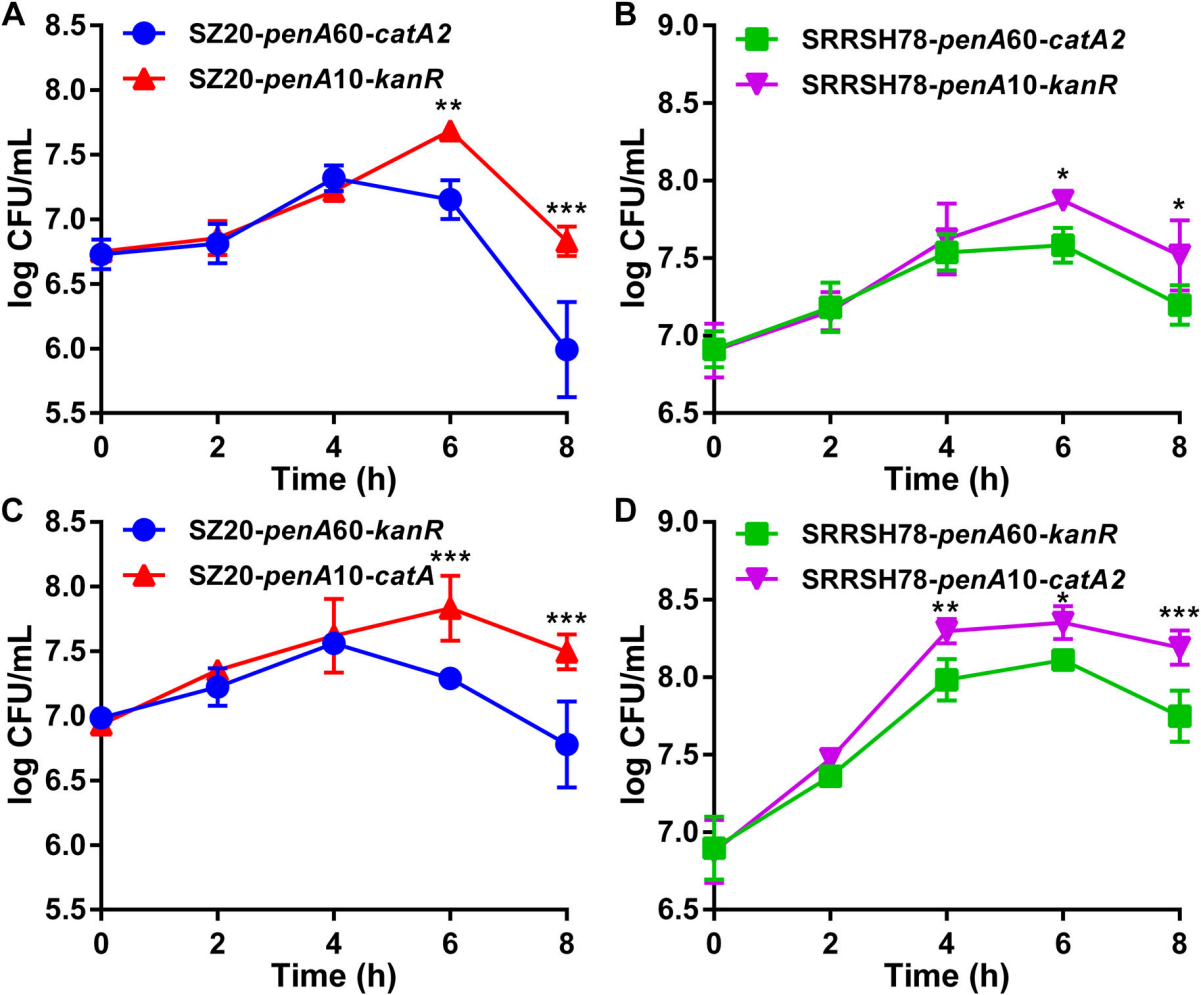
*In vitro* competition assays of the gonococcal *penA*60/*penA*10 allele exchange mutants in the presence of lithocholic acid. (A) Growth of strains SZ20-*penA*60-*catA2* and SZ20-*penA*10-*kanR* in competition in liquid culture containing 5 mg/L lithocholic acid. (B) Growth of strains SRRSH78-*penA*60-*catA2* and SRRSH78-*penA*10-*kanR* in competition in liquid culture containing 60 mg/L lithocholic acid. (C) Growth of strains SZ20-*penA*60-*kanR* and SZ20-*penA*10-*catA2* in competition in liquid culture containing 5 mg/L lithocholic acid. (D) Growth of strains SRRSH78-*penA*60-*kanR* and SRRSH78-*penA*10-*catA2* in competition in liquid culture containing 60 mg/L lithocholic acid. Competitive growth was determined by CFU counts on selective agar plates. The graphs represent the average and standard deviation of three biological independent experiments. Significant differences between the *penA*60/*penA*10 mutants at corresponding time-points were identified by Student’s two-tailed unpaired *t*-test (GraphPad Prism). *P<0.05; **P<0.01; ***P<0.001.

The *in vitro* biological fitness of the *penA* allele exchange mutants was determined during *in vitro* growth both in the presence and absence of the host antimicrobial compounds palmitic acid and lithocholic acid, which are highly abundant in the mucosal epithelia and rectum, respectively. In the absence of antimicrobial compounds, liquid growth of single strains was indistinguishable for both SZ20 (Figure 1(A)) and SRRSH78 (Figure 1(B)) when comparing the *penA* mutants expressing *penA*60 and *penA*10. To compare growth in competition, the chloramphenicol-resistance selection marker *catA2* was inserted in the *penA*60 strains and the kanamycin-resistance marker *kanR* in the *penA*10 strains and competitive growth was evaluated by CFU determination on agar plates containing chloramphenicol or kanamycin. Again, no differences in growth between the *penA*60- and *penA*10-containing mutants were observed for both SZ20 (Figure 1(C)) and SRRSH78 (Figure 1(D)). To ensure results were not affected by the respective selection markers, selection markers were changed, which gave similar results (Figure 1(E,F)). Subsequently, *penA* mutants were tested for their ability to grow in liquid cultures as single strains in the presence of high palmitic acid and lithocholic acid concentrations. Interestingly, for both strains the mutants containing *penA*60 grew significantly better at the highest permissive palmitic acid concentrations than the mutants containing *penA*10 (Figure 2(A,B)). Similar results were obtained in spot assays where *penA*60-containing mutants displayed a higher growing fraction on agar plates containing high palmitic acid concentrations (Figure 2(C)). Also, *penA*60 mutants grew significantly better in liquid culture containing the highest permissive lithocholic acid concentrations than the *penA*10-containing mutants (Figure 2(D,E)) and they showed a higher growing fraction in spot assays on plates with elevated lithocholic acid concentrations (Figure 2(F)). Subsequently, competition assays were performed with the *penA* exchange mutants for liquid growth in the presence of palmitic acid and litocholic acid using slightly lower concentrations than for the single-strain growth experiments, which was more permissive for growth of the *penA*10 strains. Interestingly, under these slightly less stressful conditions, the mutants containing *penA*10 were actually outcompeting the mutants containing *penA*60 for growth in the presence of palmitic acid, since the *penA*10-containing strains reached higher CFU counts after four to six hours growth (Figure 3). After six hours of growth, a decline in CFU counts was observed for most experiments. Similar results were obtained when selection markers were changed. Finally, competition experiments were performed in the presence of elevated lithocholic acid concentrations. Again, mutants containing *penA*10 outcompeted *penA*60-containing mutants and reached higher CFU counts after six hours of growth and CFU counts remained higher in the decline phase after eight hours incubation (Figure 4). Also under these conditions, the selection markers did not affect the final outcome, since similar differences between the *penA*60 and *penA*60 mutants were observed when selection markers were changed. Overall, these data provide a mixed picture on the impact of *penA* allele 60.001 on *in vitro* biological fitness.

### Impact of *penA* allele 60.001 on *in vivo* biological fitness in a mouse vaginal tract infection model

**Figure 5.**
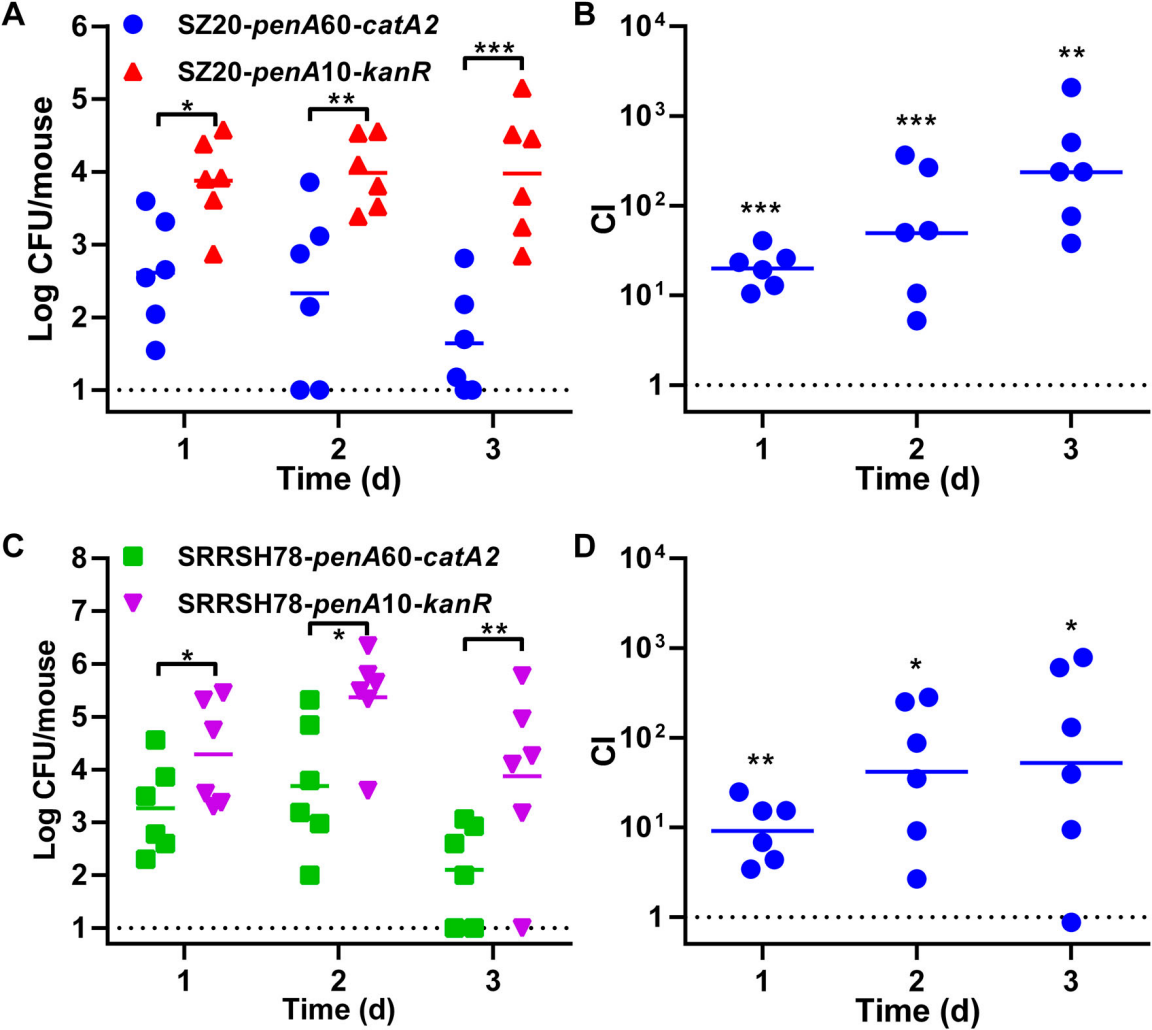
*In vivo* competition assays of the gonococcal *penA*60/*penA*10 allele exchange mutants in a mouse vaginal tract infection model. (A) Recovery of SZ20-*penA*60-*catA2* and SZ20-*penA*10-*kanR* CFUs from the mouse vaginal tract after competitive colonization. (B) Competition indices (CIs) between SZ20-*penA*60-*catA2* and SZ20-*penA*10-*kanR* based on recovered CFU counts from the mouse vaginal tract. (C) Recovery of SRRSH78-*penA*60-*catA2* and SRRSH78-*penA*10-*kanR* CFUs from the mouse vaginal tract after competitive colonization. (D) CIs between SRRSH78-*penA*60-*catA2* and SRRSH78-*penA*10-*kanR* based on recovered CFU counts from the mouse vaginal tract. The CIs were calculated as (*penA*10/*penA*60)_output_/(*penA*10/*penA*60)_input_. Significant differences in recovered CFUs between *penA*60/*penA*10 mutants and between CIs calculated for the *in vivo* mouse vaginal tract infection model and *in vitro* growth in liquid culture at corresponding time-points were identified by Student’s two-tailed unpaired *t*-test (GraphPad Prism). *P<0.05; **P<0.01; ***P<0.001.

The *in vivo* biological fitness of the *penA* allele exchange mutants was investigated by competition assays for colonization of the mouse vaginal tract. Bacterial suspensions containing equal numbers of the SZ20 or SRRSH78 mutants expressing *catA2* (*penA*60) or *kanR* (*penA*10) were used to inoculate the mouse vaginal tract and colonization was monitored for three days by daily swabbing. For both SZ20 and SRRSH78 *in vivo* competition assays, the *penA*10-containing mutants showed higher recovery of CFU counts for all three days compared with the *penA*60-containing mutants (Figure 5). Also, the calculated CI-values for all colonized mice ranged between five and two thousand, indicating that mutants containing *penA*10 outcompeted the *penA*60 mutants (Figure 5). Therefore, these data indicate that *penA* allele 60.001 has a negative impact on *in vivo* biological fitness in a mouse vaginal tract infection model.

## Discussion

The emergence and global transmission of the gonococcal FC428 clone over the past few years has become a major threat to ceftriaxone-based therapy, which is currently the last-remaining first-line treatment. Ceftriaxone resistance in the FC428 clone has been attributed to the presence of mosaic *penA* allele 60.001, although thus far its contribution to ceftriaxone resistance has been established only by association. In the current study, we showed by genetic engineering strategies that otherwise isogenic strains expressing *penA* allele 60.001 showed up to sixteen-fold higher ceftriaxone MIC values compared with strains expressing mosaic *penA* allele 10.001. Allele *penA* 10.001 is frequently encountered in gonococcal isolates in the Asia-Pacific region and has been associated with cefixime resistance or reduced susceptibility, and less abundantly with lower-level ceftriaxone resistance (up to ceftriaxone MIC = 0.25 mg/L) [8,44,45]. However, even though *penA* allele 10.001 is able to provide a major reduction in ceftriaxone susceptibility compared with many other mosaic and non-mosaic *penA* alleles, likely because it contains the I312M, F504L, N512Y and G545S polymorphisms previously associated with reduced susceptibility [37,38], *penA* allele 60.001 was still able to further reduce susceptibility over *penA* allele 10.001. The key polymorphisms in *penA* allele 60.001 that are associated with higher-level ceftriaxone resistance are A311 V and T483S. These polymorphism were also present in the ceftriaxone-resistant Australian strain A8804 [21]. This strain displayed a ceftriaxone MIC of 0.5 mg/L, which is similar to the MIC observed for the *penA* 60.001-expressing strains in our study. A previous study on the contribution of these polymorphisms showed that the introduction of individual A311 V and T483S mutations in the mosaic *penA* allele of strain 35/02 provided a 2- and 4-fold increase in ceftriaxone MICs, respectively [36]. Since that study focused on the identification of essential polymorphisms for ceftriaxone resistance in strain HO41, which contains and additional key T316P polymorphism, the combined A311 V and T483S mutations were not tested [22]. However, combining the A311 V, T316P and T483S mutations in the *penA* allele of strain 35/02 resulted in similar ceftriaxone susceptibility levels as isogenic strains expressing the HO41 *penA* allele [36].

It is often assumed that mutations providing antibiotic resistance are costly and reduce biological fitness [46,47]. Therefore, susceptible bacteria are able to outcompete resistant bacteria in the absence of antibiotic pressure, which might prevent widespread transmission of some of the most resistant strains. Indeed, it has been shown that mosaic *penA* alleles of the ceftriaxone-resistant gonococcal strains HO41 and F89 reduced biological fitness during *in vitro* liquid growth and *in vivo* in a mouse model of infection [39], which would explain why these strains have not shown widespread transmission. Interestingly, *in vivo* competition assays with isogenic strains expressing the ceftriaxone-resistant *penA* alleles of HO41 (allele 37) and F89 (allele 42) allowed for the rapid arise of compensatory mutations for the HO41 *penA* allele, but not the F89 *penA* allele for which resistance was dependent on an A501P polymorphism [17,39,48]. Importantly, for *N. gonorrhoeae* several mutations have already been described that provide antibiotic resistance and also improve biological fitness. Mutations in *mtrR* and its promoter, which alleviate repression of the MtrCDE multidrug efflux pump, are advantageous for fitness during colonization of a mouse model of infection [49,50]. Multidrug-resistant strains generally contain one or more *mtrR* mutations to increase efflux of hydrophobic or amphipathic antibiotics, while increased efflux of host-derived antimicrobial compounds such as fatty acids, bile and antimicrobial peptides allows for increased *in vivo* fitness [41,49,50]. Similarly, the 23S rRNA A2059G polymorphism is the sole mutation providing high-level azithromycin resistance and furthermore enhances *in vivo* biological fitness in a mouse vaginal tract infection model [40]. Our current results provide a more mixed picture on the impact of *penA* allele 60.001 on biological fitness. Strains expressing *penA* allele 60.001 were outcompeted by isogenic strains expressing *penA* allele 10.001 for *in vivo* colonization in a mouse model of infection and for *in vitro* liquid growth in the presence of additional stress (fatty acid/bile). However, both single-strain growth and competitive growth *in vitro* in liquid cultures in the absence of additional stress was identical between the *penA* 60.001 and *penA* 10.001 isogenic strains. Furthermore, during single-strain *in vitro* growth experiments in the presence of additional stress, which was at a higher stress level than during the competitive growth experiments, the *penA* 60.001 strains actually grew better. Therefore, it seems that *penA* allele 60.001 actually allows *N. gonorrhoeae* to grow at a higher stress level, even though it negatively impacts competitive growth at a lower stress level. Translation of these results to fitness during colonization of the human host will be difficult. Whether strains containing *penA* allele 60.001 will show reduced fitness in the human host might really be dependent on the combination of stresses encountered, but given that the FC428 clone has already shown global transmission, its fitness defects in the human host are likely very limited.

In conclusion, here we showed that *penA* allele 60.001 of the ceftriaxone-resistant gonococcal FC428 clone reduces ceftriaxone susceptibility by eight- to sixteen-fold compared with mosaic *penA* allele 10.001. Further analysis of the impact of *penA* allele 60.001 on biological fitness provided a mixed picture where *penA* 60.001 negatively impacts *in vivo* fitness in a mouse vaginal tract infection model, while *in vitro* liquid growth in the absence of additional stress seemed unaffected. Therefore, the negative impact of *penA* allele 60.001 on biological fitness might not be very severe, which would explain the successful global transmission of the FC428 clone in recent years.

## References

[1] Unemo M, Lahra MM, Cole M, et al. World Health Organization Global Gonococcal Antimicrobial Surveillance Program (WHO GASP): review of new data and evidence to inform international collaborative actions and research efforts. Sex Health. 2019 Sep;16(5):412–425. doi: 10.1071/SH19023 31437420 PMC7035961

[2] Edwards JL, Apicella MA. The molecular mechanisms used by *Neisseria gonorrhoeae* to initiate infection differ between men and women. Clin Microbiol Rev. 2004 Oct;17(4):965–981. doi: 10.1128/CMR.17.4.965-981.2004 15489357 PMC523569

[3] Farley TA, Cohen DA, Elkins W. Asymptomatic sexually transmitted diseases: the case for screening. Prev Med. 2003 Apr;36(4):502–509. doi: 10.1016/S0091-7435(02)00058-0 12649059

[4] Hook EW, Bernstein K. Kissing, saliva exchange, and transmission of *Neisseria gonorrhoeae*. Lancet Infect Dis. 2019 Oct;19(10):e367–e369. doi: 10.1016/S1473-3099(19)30306-8 31324518 PMC6764880

[5] Unemo M, Shafer WM. Antimicrobial resistance in *Neisseria gonorrhoeae* in the 21st century: past, evolution, and future. Clin Microbiol Rev. 2014 Jul;27(3):587–613. doi: 10.1128/CMR.00010-14 24982323 PMC4135894

[6] Fifer H, Cole M, Hughes G, et al. Sustained transmission of high-level azithromycin-resistant *Neisseria gonorrhoeae* in England: an observational study. Lancet Infect Dis. 2018 May;18(5):573–581. doi: 10.1016/S1473-3099(18)30122-1 29523496

[7] Katz AR, Komeya AY, Kirkcaldy RD, et al. Cluster of *Neisseria gonorrhoeae* isolates with high-level azithromycin resistance and decreased ceftriaxone susceptibility, Hawaii, 2016. Clin Infect Dis. 2017 Sep 15;65(6):918–923. doi: 10.1093/cid/cix485 28549097 PMC6748320

[8] Yan J, Xue J, Chen Y, et al. Increasing prevalence of *Neisseria gonorrhoeae* with decreased susceptibility to ceftriaxone and resistance to azithromycin in Hangzhou, China (2015-17). J Antimicrob Chemother. 2019 Oct 16;74(1):29–37. 30329062 10.1093/jac/dky412

[9] Kong FYS, Horner P, Unemo M, et al. Pharmacokinetic considerations regarding the treatment of bacterial sexually transmitted infections with azithromycin: a review. J Antimicrob Chemother. 2019 May 1;74(5):1157–1166. doi: 10.1093/jac/dky548 30649333

[10] Ison CA, Town K, Obi C, et al. Decreased susceptibility to cephalosporins among gonococci: data from the gonococcal resistance to Antimicrobials Surveillance Programme (GRASP) in England and Wales, 2007-2011. Lancet Infect Dis. 2013 Sep;13(9):762–768. doi: 10.1016/S1473-3099(13)70143-9 23764300

[11] Shimuta K, Watanabe Y, Nakayama S, et al. Emergence and evolution of internationally disseminated cephalosporin-resistant *Neisseria gonorrhoeae* clones from 1995 to 2005 in Japan. BMC Infect Dis. 2015 Sep 17;15:378. doi: 10.1186/s12879-015-1110-x 26381611 PMC4574456

[12] Yin YP, Han Y, Dai XQ, et al. Susceptibility of *Neisseria gonorrhoeae* to azithromycin and ceftriaxone in China: A retrospective study of national surveillance data from 2013 to 2016. PLoS Med. 2018 Feb;15(2):e1002499. doi: 10.1371/journal.pmed.1002499 29408881 PMC5800545

[13] Chen MY, Stevens K, Tideman R, et al. Failure of 500 mg of ceftriaxone to eradicate pharyngeal gonorrhoea, Australia. J Antimicrob Chemother. 2013 Jun;68(6):1445–1447. doi: 10.1093/jac/dkt017 23390207

[14] Poncin T, Fouere S, Braille A, et al. Multidrug-resistant *Neisseria gonorrhoeae* failing treatment with ceftriaxone and doxycycline in France, November 2017. Euro Surveill. 2018 May;23(21):1800264. doi: 10.2807/1560-7917.ES.2018.23.21.1800264 29845928 PMC6152217

[15] Tapsall J, Read P, Carmody C, et al. Two cases of failed ceftriaxone treatment in pharyngeal gonorrhoea verified by molecular microbiological methods. J Med Microbiol. 2009 May;58(Pt 5):683–687. doi: 10.1099/jmm.0.007641-0 19369534

[16] Unemo M, Golparian D, Hestner A. Ceftriaxone treatment failure of pharyngeal gonorrhoea verified by international recommendations, Sweden, July 2010. Euro Surveill. 2011 Feb 10;16(6):19792. 21329645

[17] Unemo M, Golparian D, Nicholas R, et al. High-level cefixime- and ceftriaxone-resistant *Neisseria gonorrhoeae* in France: novel penA mosaic allele in a successful international clone causes treatment failure. Antimicrob Agents Chemother. 2012 Mar;56(3):1273–1280. doi: 10.1128/AAC.05760-11 22155830 PMC3294892

[18] Unemo M, Golparian D, Potocnik M, et al. Treatment failure of pharyngeal gonorrhoea with internationally recommended first-line ceftriaxone verified in Slovenia, September 2011. Euro Surveill. 2012 Jun 21;17(25):20200. 22748003

[19] Camara J, Serra J, Ayats J, et al. Molecular characterization of two high-level ceftriaxone-resistant *Neisseria gonorrhoeae* isolates detected in Catalonia, Spain. J Antimicrob Chemother. 2012 Aug;67(8):1858–1860. doi: 10.1093/jac/dks162 22566592

[20] Deguchi T, Yasuda M, Hatazaki K, et al. New clinical strain of *Neisseria gonorrhoeae* with decreased susceptibility to ceftriaxone, Japan. Emerg Infect Dis. 2016 Jan;22(1):142–144. doi: 10.3201/eid2201.150868 26689442 PMC4696695

[21] Lahra MM, Ryder N, Whiley DM. A new multidrug-resistant strain of *Neisseria gonorrhoeae* in Australia. N Engl J Med. 2014 Nov 6;371(19):1850–1851. doi: 10.1056/NEJMc1408109 25372111

[22] Ohnishi M, Golparian D, Shimuta K, et al. Is *Neisseria gonorrhoeae* initiating a future era of untreatable gonorrhea?: detailed characterization of the first strain with high-level resistance to ceftriaxone. Antimicrob Agents Chemother. 2011 Jul;55(7):3538–3545. doi: 10.1128/AAC.00325-11 21576437 PMC3122416

[23] Nakayama S, Shimuta K, Furubayashi K, et al. New ceftriaxone- and multidrug-resistant *Neisseria gonorrhoeae* strain with a novel mosaic *penA* gene isolated in Japan. Antimicrob Agents Chemother. 2016 Jul;60(7):4339–4341. doi: 10.1128/AAC.00504-16 27067334 PMC4914677

[24] Lee K, Nakayama SI, Osawa K, et al. Clonal expansion and spread of the ceftriaxone-resistant *Neisseria gonorrhoeae* strain FC428, identified in Japan in 2015, and closely related isolates. J Antimicrob Chemother. 2019 Jul 1;74(7):1812–1819. doi: 10.1093/jac/dkz129 31002306

[25] Chen SC, Han Y, Yuan LF, et al. Identification of internationally disseminated ceftriaxone-resistant *Neisseria gonorrhoeae* strain FC428, China. Emerg Infect Dis. 2019 Jul;25(7):1427–1429. doi: 10.3201/eid2507.190172 30900979 PMC6590750

[26] Yang F, Zhang H, Chen Y, et al. Detection and analysis of two cases of the internationally spreading ceftriaxone-resistant *Neisseria gonorrhoeae* FC428 clone in China. J Antimicrob Chemother. 2019 Dec 1;74(12):3635–3636. doi: 10.1093/jac/dkz384 31504585

[27] Yuan Q, Li Y, Xiu L, et al. Identification of multidrug-resistant *Neisseria gonorrhoeae* isolates with combined resistance to both ceftriaxone and azithromycin, China, 2017-2018. Emerg Microbes Infect. 2019;8(1):1546–1549. doi: 10.1080/22221751.2019.1681242 31661379 PMC6830194

[28] Terkelsen D, Tolstrup J, Johnsen CH, et al. Multidrug-resistant *Neisseria gonorrhoeae* infection with ceftriaxone resistance and intermediate resistance to azithromycin, Denmark, 2017. Euro Surveill. 2017 Oct;22(42):1700659. doi: 10.2807/1560-7917.ES.2017.22.42.17-00659 PMC5710115 29067905

[29] Lefebvre B, Martin I, Demczuk W, et al. Ceftriaxone-resistant *Neisseria gonorrhoeae*, Canada, 2017. Emerg Infect Dis. 2018 Feb;24(2):381–383. doi: 10.3201/eid2402.171756 29131780 PMC5782888

[30] Lahra MM, Martin I, Demczuk W, et al. Cooperative recognition of internationally disseminated ceftriaxone-resistant *Neisseria gonorrhoeae* strain. Emerg Infect Dis. 2018 Apr;24(4):735–740. doi: 10.3201/eid2404.171873 29553335 PMC5875269

[31] Golparian D, Rose L, Lynam A, et al. Multidrug-resistant *Neisseria gonorrhoeae* isolate, belonging to the internationally spreading Japanese FC428 clone, with ceftriaxone resistance and intermediate resistance to azithromycin, Ireland, August 2018. Euro Surveill. 2018 Nov;23(47):1800617. doi: 10.2807/1560-7917.ES.2018.23.47.1800617 30482267 PMC6341943

[32] Eyre DW, Town K, Street T, et al. Detection in the United Kingdom of the *Neisseria gonorrhoeae* FC428 clone, with ceftriaxone resistance and intermediate resistance to azithromycin, October to December 2018. Euro Surveill. 2019 Mar;24(10):1900147. doi: 10.2807/1560-7917.ES.2019.24.10.1900147 30862336 PMC6415501

[33] Eyre DW, Sanderson ND, Lord E, et al. Gonorrhoea treatment failure caused by a *Neisseria gonorrhoeae* strain with combined ceftriaxone and high-level azithromycin resistance, England, February 2018. Euro Surveill. 2018 Jul;23(27):1800323. doi: 10.2807/1560-7917.ES.2018.23.27.1800323 29991383 PMC6152157

[34] Chen SC, van der Veen S, Yin YP. Widespread transmission of the ceftriaxone-resistant *Neisseria gonorrhoeae* FC428 clone in China. Submitted. 10.1093/jac/dkaa196 32473014

[35] Yan J, Chen Y, Yang F, et al. High incidence of the ceftriaxone-resistant *Neisseria gonorrhoeae* FC428 clone in Hangzhou, China. Submitted. 10.1093/jac/dkaa526 33406237

[36] Tomberg J, Unemo M, Ohnishi M, et al. Identification of amino acids conferring high-level resistance to expanded-spectrum cephalosporins in the *penA* gene from *Neisseria gonorrhoeae* strain H041. Antimicrob Agents Chemother. 2013 Jul;57(7):3029–3036. doi: 10.1128/AAC.00093-13 23587946 PMC3697319

[37] Singh A, Tomberg J, Nicholas RA, et al. Recognition of the beta-lactam carboxylate triggers acylation of *Neisseria gonorrhoeae* penicillin-binding protein 2. J Biol Chem. 2019 Sep 20;294(38):14020–14032. doi: 10.1074/jbc.RA119.009942 31362987 PMC6755799

[38] Tomberg J, Unemo M, Davies C, et al. Molecular and structural analysis of mosaic variants of penicillin-binding protein 2 conferring decreased susceptibility to expanded-spectrum cephalosporins in *Neisseria gonorrhoeae*: role of epistatic mutations. Biochemistry. 2010 Sep 21;49(37):8062–8070. doi: 10.1021/bi101167x 20704258 PMC2939205

[39] Vincent LR, Kerr SR, Tan Y, et al. *In vivo*-selected compensatory mutations restore the fitness cost of mosaic *penA* alleles that confer ceftriaxone resistance in *Neisseria gonorrhoeae*. MBio. 2018 Apr 3;9(2):e01905-17. doi: 10.1128/mBio.01905-17 PMC5885032 29615507

[40] Zhang J, van der Veen S. *Neisseria gonorrhoeae* 23S rRNA A2059G mutation is the only determinant necessary for high-level azithromycin resistance and improves *in vivo* biological fitness. J Antimicrob Chemother. 2019 Feb 1;74(2):407–415. doi: 10.1093/jac/dky438 30376120

[41] Wang S, Xue J, Lu P, et al. Gonococcal MtrE and its surface-expressed Loop 2 are immunogenic and elicit bactericidal antibodies. J Infect. 2018 Sep;77(3):191–204. doi: 10.1016/j.jinf.2018.06.001 29902495

[42] Wang Z, Wang X, Lu P, et al. Identification and characterization of the *Neisseria gonorrhoeae* MscS-like mechanosensitive channel. Infect Immun. 2018 Jun;86(6):e00090-18. doi: 10.1128/IAI.00090-18 29581189 PMC5964504

[43] Jerse AE, Sharma ND, Simms AN, et al. A gonococcal efflux pump system enhances bacterial survival in a female mouse model of genital tract infection. Infect Immun. 2003 Oct;71(10):5576–5582. doi: 10.1128/IAI.71.10.5576-5582.2003 14500476 PMC201053

[44] Ito M, Deguchi T, Mizutani KS, et al. Emergence and spread of *Neisseria gonorrhoeae* clinical isolates harboring mosaic-like structure of penicillin-binding protein 2 in Central Japan. Antimicrob Agents Chemother. 2005 Jan;49(1):137–143. doi: 10.1128/AAC.49.1.137-143.2005 15616287 PMC538884

[45] Lee H, Suh YH, Lee S, et al. Emergence and spread of cephalosporin-resistant *Neisseria gonorrhoeae* with mosaic *penA* alleles, South Korea, 2012-2017. Emerg Infect Dis. 2019 Mar;25(3):416–424. doi: 10.3201/eid2503.181503 30789143 PMC6390772

[46] Andersson DI, Levin BR. The biological cost of antibiotic resistance. Curr Opin Microbiol. 1999 Oct;2(5):489–493. doi: 10.1016/S1369-5274(99)00005-3 10508723

[47] Nilsson AI, Zorzet A, Kanth A, et al. Reducing the fitness cost of antibiotic resistance by amplification of initiator tRNA genes. Proc Natl Acad Sci U S A. 2006 May 2;103(18):6976–6981. doi: 10.1073/pnas.0602171103 16636273 PMC1459004

[48] Tomberg J, Fedarovich A, Vincent LR, et al. Alanine 501 mutations in penicillin-binding protein 2 from *Neisseria gonorrhoeae*: structure, mechanism, and effects on cephalosporin resistance and biological fitness. Biochemistry. 2017 Feb 28;56(8):1140–1150. doi: 10.1021/acs.biochem.6b01030 28145684 PMC5502787

[49] Warner DM, Folster JP, Shafer WM, et al. Regulation of the MtrC-MtrD-MtrE efflux-pump system modulates the in vivo fitness of *Neisseria gonorrhoeae*. J Infect Dis. 2007 Dec 15;196(12):1804–1812. doi: 10.1086/522964 18190261

[50] Warner DM, Shafer WM, Jerse AE. Clinically relevant mutations that cause derepression of the *Neisseria gonorrhoeae* MtrC-MtrD-MtrE efflux pump system confer different levels of antimicrobial resistance and *in vivo* fitness. Mol Microbiol. 2008 Oct;70(2):462–478. doi: 10.1111/j.1365-2958.2008.06424.x 18761689 PMC2602950

